# Evaluation of Functional Erythropoietin Receptor Status in Skeletal Muscle In Vivo: Acute and Prolonged Studies in Healthy Human Subjects

**DOI:** 10.1371/journal.pone.0031857

**Published:** 2012-02-22

**Authors:** Britt Christensen, Carsten Lundby, Niels Jessen, Thomas S. Nielsen, Poul F. Vestergaard, Niels Møller, Henriette Pilegaard, Steen B. Pedersen, John J. Kopchick, Jens Otto L. Jørgensen

**Affiliations:** 1 Department of Endocrinology and Internal Medicine, NBG/THG, Aarhus University Hospital, Aarhus, Denmark; 2 Medical Research Laboratories, University of Aarhus, Aarhus, Denmark; 3 Center for Integrative Human Physiology (ZIHP), Institute of Physiology, University of Zürich, Zürich, Switzerland; 4 Department of Clinical Pharmacology, Aarhus University Hospital, Aarhus, Denmark; 5 Edison Biotechnology Institute, Department of Biomedical Sciences, Ohio University, Athens, Ohio, United States of America; 6 Department of Biomedical Sciences, Ohio University, Athens, Ohio, United States of America; 7 Centre of Inflammation and Metabolism and Copenhagen Muscle Research Centre, August Krogh Building, Department of Biology, University of Copenhagen, Copenhagen, Denmark; Universidad Europea de Madrid, Spain

## Abstract

**Background:**

Erythropoietin receptors have been identified in human skeletal muscle tissue, but downstream signal transduction has not been investigated. We therefore studied in vivo effects of systemic erythropoietin exposure in human skeletal muscle.

**Methodology/Principal Findings:**

The protocols involved 1) acute effects of a single bolus injection of erythropoietin followed by consecutive muscle biopsies for 1–10 hours, and 2) a separate study with prolonged administration for 16 days with biopsies obtained before and after. The presence of erythropoietin receptors in muscle tissue as well as activation of Epo signalling pathways (STAT5, MAPK, Akt, IKK) were analysed by western blotting. Changes in muscle protein profiles after prolonged erythropoietin treatment were evaluated by 2D gel-electrophoresis and mass spectrometry. The presence of the erythropoietin receptor in skeletal muscle was confirmed, by the M20 but not the C20 antibody. However, no significant changes in phosphorylation of the Epo-R, STAT5, MAPK, Akt, Lyn, IKK, and p70S6K after erythropoietin administration were detected. The level of 8 protein spots were significantly altered after 16 days of rHuEpo treatment; one isoform of myosin light chain 3 and one of desmin/actin were decreased, while three isoforms of creatine kinase and two of glyceraldehyd-3-phosphate dehydrogenase were increased.

**Conclusions/Significance:**

Acute exposure to recombinant human erythropoietin is not associated by detectable activation of the Epo-R or downstream signalling targets in human skeletal muscle in the resting situation, whereas more prolonged exposure induces significant changes in the skeletal muscle proteome. The absence of functional Epo receptor activity in human skeletal muscle indicates that the long-term effects are indirect and probably related to an increased oxidative capacity in this tissue.

## Introduction

Erythropoietin (Epo) is the main regulator of erythropoiesis [1]. The primary site for Epo production is the kidney, where it is produced in a hypoxia-dependent manner. However, small amounts are also produced in the liver and brain [2]. Epo binds to a specific receptor (Epo-R), that belongs to the cytokine receptor superfamily and activates the JAK/STAT, PI3-kinase, NF-κB/IKK, and/or the Ras/MAP kinase pathways [1], [3], [4]. Through these pathways Epo exerts anti-apoptotic effects during the later stages of erythroid progenitor cell development in the bone marrow, by decreasing the rate of cell death and hence inducing these cells to proliferate and mature [1].

Epo-Rs have been identified on a variety of different cell types including renal, endothelial, vascular smooth muscle, gastric mucosal, and Leydig cells, as well as cells of the placenta, certain cancer cells, cardiomyocytes, astrocytes, and neurons [2], [5]–[10]. The main biological function of Epo in these cells is to facilitate proliferation, angiogenesis, and cytoprotection [2], [9], [11]. Furthermore, Epo-Rs are expressed in vitro on murine myoblasts and primary satellite cells, both of which exhibit a proliferative response to Epo stimulation [12]. Recently, the Epo-R was also discovered on human skeletal muscle cells [13], [14]; however, the physiological role of Epo in this tissue remains uncertain [15].

Several studies have investigated changes in mRNA levels of pertinent proteins and structural changes in muscle after recombinant human Epo (rHuEpo) administration with conflicting results [13], [16], [17]. Thus, even though the Epo-R has been identified in human skeletal muscle tissue, its role remains incompletely understood.

Investigations of the activation of the signalling cascades related to the Epo-R, could give insight into the physiological role of the Epo-R in skeletal muscle tissue. To our knowledge, no previous studies have analysed Epo induced intracellular signalling pathways in human skeletal muscle in vivo. We therefore investigated the activation of a variety of molecules involved in signalling from the Epo-R (STAT5, p38-MAPK, Akt, Lyn, IKK, and p70S6K) and gene transcripts (SOCS-3) in response to acute stimulation of the Epo-R by rHuEpo. Lyn is a non-receptor protein tyrosine kinase, which acts as a docking protein that is pre-associated with the Epo-R and bind to the Epo-R and Jak2 [18]. Lyn mediates the phosphorylation of the Epo-R and activation of the signalling cascades STAT5, PI3-K and NF-κB [18]–[20]. The main signalling pathways through which Epo signals are STAT5, MAPK, PI3-K/akt, and NF-κB/IKK [1], [2], [4], each of these pathways were investigated here. Epo-R signalling is reversibly inhibited by SOCS-3, wherefore its gene transcript was measured [21]. Furthermore, IGF-I expression was measured to rule out any GH induced activation of the signalling cascades of interest. Moreover, we also identified changes in human muscle proteome following prolonged Epo administration. In the current study two different doses of rHuEpo was investigated. In study A, a dose of 15,000 IU was administrated, which is comparable to the doses used to treat patients with end-stage renal disease. In study B, an even higher dose (400 IU/kg∼32,000 IU per subject), comparable to the dose employed to treat patients with stroke, was used.

Based on the presence of the Epo-R in skeletal muscle tissue, we hypothesized that rHuEpo treatment would lead to activation of STAT5, p38-MAPK, Akt, Lyn, IKK, and p70S6K downstream of the Epo-R, which would lead to changes in the skeletal muscle protein content.

## Methods

### Subjects and ethical approval

#### Acute studies (A and B)

In study A, eight healthy male subjects (27±7 yr, 180±4 cm, 83±7 kg, mean ± SE) were included, all of whom provided a written informed consent to participate in the study, which was approved by the local human ethical committee of Copenhagen and Frederiksberg (KF 01-269-637), in adherence to the declaration of Helsinki. Data related to changes in mRNA content among these subjects have previously been published [13].

In study B, ten healthy young men (23±0.7 yr, 180±2 cm, 76.6±2.2 kg, mean ± SE) were enrolled. All subjects provided a written informed consent to participate in the study, which was approved by the local human ethical committee of Central Denmark Region (M-2008-0016), in adherence to the declaration of Helsinki.

#### Prolonged study (C)

Eight healthy male volunteers were included (25±4 yr, 183±6 cm, 79±7 kg, mean ± SE). All subjects provided a written informed consent to participate in the study, which was approved by the local human ethical committee of Copenhagen and Frederiksberg, Denmark (KF 01 269 637), in adherence to the declaration of Helsinki. Results describing basic serum hematological changes after Epo administration to these subjects have already been published [22].

### Experimental design

#### Acute studies (A and B)

Study A was performed in a double-blind, randomised, placebo-controlled, crossover design. The subjects arrived fasting (from 10 pm the day before) at the lab and were served a light standardized breakfast adjusted for body weight and activity level; a blood sample and the first biopsy (pre) were collected 2 hours later after resting in the supine position. The biopsies were collected from m. vastus lateralis and taken before (Pre) and 2 h, 4 h, 6 h, and 10 h post I.V. administration of either rHuEpo (15.000 IU, NeoRecormon, Roche) or placebo (saline). Biopsies were immediately frozen in liquid nitrogen, and stored at −80°C until further analysis. After the 6 h biopsy, the subjects were served a standardized meal (same on both experimental days). Blood samples were taken at the same time-points as the biopsies, centrifuged at 2500× g for 15 minutes, and stored at −20°C until analysed. Biopsies from before injection of rHuEpo/placebo and 2 h, 4 h, and 6 h post, were used for protein extraction and western blotting. The 10 h post biopsy was used for mRNA quantification.

Study B had a single-blind, randomised, placebo-controlled, cross-over design with a 14-day wash-out period in-between. Before enrolment, the subjects were examined by a medical doctor to ensure general health and standard blood analysis (haematology, organ markers (LDH, ALAT, bilirubin and basic phosphatase), and electrolyte balance) was performed. The subjects were examined on two occasions: 1) i.v. treatment with 400 IU/kg Eprex (Epoietin alpha) or 2) placebo (saline), both administered at t = 0 min. The subjects arrived fasting (from 10pm the evening before, water allowed) at the lab in the morning. Muscle biopsies were collected from m. vastus lateralis and taken one hour (t = 60 min) after Epo/saline administration. The biopsies were immediately frozen in liquid nitrogen and stored at −80°C until further analysis. Serum and plasma were collected 4 h post treatment, centrifuged and stored at −20°C.

#### Prolonged study (C)

Muscle biopsies from m. vastus lateralis were collected approximately 1 week before the first Epo injection. Epo (epoetin β; NeoRecormon, Roche, Mannheim, Germany) was injected every second day (day 0, 2, 4, 6, 8, 10, 12, 14) subcutaneously at a dose of 5000 IU. A second muscle biopsy was collected on day 16. Muscle biopsies were frozen immediately in liquid nitrogen and stored at −80°C until further analysis was performed.

### Analysis in plasma/serum

#### Study A

Plasma concentrations of insulin and GH were measured in duplicates by ELISA (Electra-Box diagnostica, Tyresö, Sweden; IBL-Hamburg, Germany; BioSource, Nivelles, Belgium), as previously described [13].

#### Study B

Insulin and GH were analysed by commercial time-resolved immunefluorometric assays (TR-IFMA; AutoDELFIA, PerkinElmer, Turku, Finland) (Insulin: intra-assay CV 3.4% and inter-assay CV 3.8%, GH: intra-assay CV<8% and inter-assay CV<10%).

### Cell signaling analysis

#### Protein purification

Proteins were purified from the biopsies (30–50 mg) by homogenization on ice with a polytron in homogenization buffer (20 mM Tris HCL, 50 mM NaF, 5 mM tetrasodium pyrophosphate, 270 mM sucrose, 1% (v/v) Triton-X100, 1 mM EDTA, 1 mM EGTA, 10 mM glycerolphosphat, 2 mM DTT, 50 µg/ml soybean trypsin inhibitor, 4 µg/ml leupeptin, 100 µM benzamidine, and 500 µM PMSF, pH = 7.4). The homogenate was left on ice for 30 min with occasional vortexing, before being centrifuged at 14000 g at 4°C for 20 min. The supernatant was collected, frozen in liquid nitrogen, and stored at −80°C until analyses were performed. Protein concentration was determined by the Bradford assay (Protein Assay, #500-0006, Bio Rad laboratories Inc, CA, USA. Albumin standard, Thermo Scientific, IL, USA. Victor 3, 1420 multilabel counter, Perkin Elmer).

#### Western blot analysis

The protein fraction was analysed for phosphorylation of Epo-R, Lyn, STAT5, Akt, p70S6-kinase, MAPK and for total Epo-R. Primary antibodies were as follows: anti-phospho-Epo-R(Tyr456) (Santa Cruz, #sc20236), anti-Lyn (Cell signalling, #2732), anti-phospho-Lyn(Tyr507) (Cell signalling, #2731), anti-STAT5 (Cell signalling, #9310), anti-phospho-STAT5(Tyr694) (Cell signalling, #9351), anti-Akt/PKB (Cell signalling, #9272), anti-phospho-Akt/PKB(Ser473) (Cell signalling, #9271), anti-phospho-Akt/PKB(Thr308) (Cell signalling, #9275), anti-p70S6 kinase (Cell signalling, #9202), anti-phospho-p70S6 kinase(Thr389) (Cell signalling, #9205), anti-p38-MAP kinase (Cell signalling, #9212), anti-phospho-p38-MAP kinase (Thr180/Thr182) (Cell signalling, #9211), anti-IKKα (Cell signalling, #2682), anti-phospho-IKKα/β(Ser176/180) (Cell signalling, #2697), anti-Epo-R (M20) (Santa Cruz, #sc697), anti-Epo-R (C20) (Santa Cruz, #sc695), and anti-β-actin (Abcam, #ab8227). Donkey anti-rabbit IgG horseradish peroxidise (HRP) was used as secondary antibody (Amersham, #NA934). Briefly, western blotting was performed as follows; 20–30 µg of protein was loaded onto a 4–12% SDS Criterion Gel (BioRad, Hercules, CA, USA), followed by electro blotting onto a nitrocellulose or PVDF membrane. Membranes were blocked with blocking buffer (5% BSA, 0.01% NaN_3_ in TBS buffer) before primary antibody was added overnight at 4°C. Following several washes, the membrane was incubated with the secondary antibody for 60 min at room temperature. The protein of interest was detected by a chemiluminescence detection system (Super Signal West Dura Extended duration substrate, Pierce cat.no. 34075) and visualized using an image system (UPV BioImaging systems). The PVDF membranes were stripped after visualization of the phospho-antibodies and re-incubated with the total antibodies. Membranes were stripped (62,5 mM Tris HCL ph 6.8, 2% SDS, 73,75 ml dH_2_O) for 1 h at 55°C.

#### Real-time PCR

Skeletal muscle (30 mg) samples were homogenized in TriZol reagent (Gibco BRL, Life Technologies, Roskilde, Denmark) added DNase and proteinaseK and total RNA was extracted following the manufacture's protocol. RNA was quantified by measuring absorbance at 260 and 280 nm using a NanoDrop 8000 (NanoDrop products, Bancroft, DE USA), and the inclusion criteria was a ratio ≥1.8. Finally, the integrity of the RNA was checked by visual inspection of the two ribosomal RNAs, 18 S and 28 S, on an agarose gel.

For real-time reverse transcriptase PCR, complementary DNA was constructed using random hexamer primers as described by the manufacturer (Verso cDNA kit, Abgene, Epsom, UK). Then KAPA SYBR FAST qPCR mastermix (Kapa Biosystems, Inc. Woburn, MA, USA) and the following primer pairs were added:

SOCS3 primers: 5′-GCCCTTTGCGCCCTTT-3′ and 5′-CGGCCACCTGGACTCCTATGA-3′, IGF-I primers (all four isoforms): 5′-GACAGGGGCTTTATTTCAAC-3′ and 5′-CTCCAGCCTCCTTAGATCAC-3′, β-actin: 5′-TGTGCCCATCTACGAGGGGTA-TGC-3′ and 5′-GGTACATGGTGGTG­CCGCCA­GACA-3′. Real-time quantification of genes was performed using an ICycler from Bio-Rad (Bio-Rad Laboratories, Hercules, CA, USA). cDNA with specific primers amplified in separate tubes, and the increase in fluorescence was measured in real time. The threshold cycle was calculated, and the relative gene expression was calculated as target gene (X0) to β-actin (R0) ratio in each sample before amplification using X0/R0 = kx1/((2*DCt)) essentially as described in the User Bulletin no. 2, 1997, from Perkin-Elmer. All samples were amplified in duplicate. A similar set-up was used for negative controls, except that the reverse transcriptase was omitted and no PCR products were detected under these conditions.

### Proteomics analysis

#### Sample preparation

Before 2D electrophoresis, the muscle biopsies were homogenized with a mechanical blender and sonication in sample buffer (7 M urea, 2 M thiourea, 1% w/v SB 3–10, 3% w/v CHAPS, 0.25% v/v Bio-lyte 3/10 ampholytes (Bio-Rad Laboratories Inc., Hercules, CA)) added 1.5% v/v protease inhibitor cocktail (Sigma, St Louis, MO). The total protein content of each sample was determined using the Bradford method. Muscle sample volumes containing 300 µg of protein were diluted in sample buffer (7 M urea, 2 M thiourea, 1% w/v SB 3–10, 3% w/v CHAPS, 0.25% v/v Bio-Lyte 3/10 ampholytes (Bio-Rad Laboratories Inc., Hercules, CA)) containing 1.5% v/v protease inhibitor cocktail (Sigma, St Louis, MO). Disulfide bonds were reduced by addition of tributylphosphine and sulfhydryl groups were alkylated with iodoacetamide.

#### Two-dimensional gel electrophoresis (2DE)

For the first dimension, diluted and treated samples were loaded onto IPG strips (isoelectric point (pI) 3–10 linear, Bio-Rad) and passively rehydrated for two hours at room temperature. Then, strips were placed into a PROTEAN IEF cell (Bio-Rad) for isoelectric focusing consisting of 12 h of active rehydration at 250 V followed by separation at 4000 V for 60000 V h. The strips were then equilibrated for 45 min in equilibration buffer (0.375 M Tris-HCl pH 8.8, 6 M urea, 2% w/v SDS, and 20% v/v glycerol) and loaded on 15% polyacrylamide gels. SDS-PAGE was run in a PROTEAN II XL cell (Bio-Rad) at 25 mA per gel and 270 V×h. Gels were fixed (40% ethanol, 2% acetic acid, 0.005% w/v SDS), washed three times (2% acetic acid, 0.005% w/v SDS), stained using SYPRO Orange (Molecular Probes, Inc., Eugene, OR), and finally scanned in a PharosFX Plus Molecular Imager (Bio-Rad) with an excitation wavelength of 488 nm and emission detected at 605 nm.

#### Image analysis

Protein spots in the gels were matched using the image analysis software PDQuest Advanced v. 8.0 (Bio-Rad) and all matches were confirmed manually. Protein spot intensities were normalized to the total image density in each gel, which depended on the total protein content of the sample.

#### Mass spectrometry (MS)

Protein spots displaying significant (p<0.05) intensity changes at the time-points studied were manually excised from the gels and sent to Protea Biosciences Inc. Morgantown, WV for analysis by mass spectrometry (MS) and tandem-MS (MS/MS) using matrix assisted laser desorption/ionization-time of flight (MALDI-TOF) and MALDI-TOF-TOF.

#### Protein identification

Protein identities obtained by Protea Biosciences were verified or revised using the MS and MS/MS data and the online software Mascot. Search parameters included the following: MS: database: NCBInr; taxonomy: *Homo sapiens*; enzyme: trypsin; missed cleavages allowed: 1; fixed modifications: none or carbamidomethyl (C); protein mass: none; peptide tolerance: ±0.1 to 1.2 Da; mass values: MH+; monoisotopic/average: monoisotopic. Tandem MS: database: NCBInr; taxonomy: *Homo sapiens*; enzyme: trypsin; missed cleavages allowed: 1; fixed modifications: none or carbamidomethyl (C); Quantitation: none; peptide tolerance: ±0.1 to 1.2 Da; MS/MS tolerance: ±0.1 to 0.6 Da; Peptide charge: 1+; monoisotopic/average: monoisotopic; Precursor m/z: none; Instrument: MALDI-TOF-TOF.

### Statistics

The level of significance was in all statistical analyses set to p<0.05. All results are expressed as mean ± SEM.

#### Study A

Differences in western-blot analysis and insulin and GH serum levels were detected using a two-way ANOVA followed by Tukey's multiple comparison tests, after checking for equal variance. Data that were not normally distributed were log transformed before analysis in order to pass normality test (Shapiro-Wilk). IGF-I mRNA levels were analyzed by a paired Student's t-test, after checking for normality (Shapiro-Wilk). The effect of Epo on SOCS3 mRNA content was tested by Wilcoxon signed rank test, as the data was not normally distributed.

#### Study B

For detection of differences between the placebo and rHuEpo treatment, a paired Student's t-test was used, after checking for normality (tested by Shapiro-Wilk). Data that did not obtain normality were log transformed.

#### Study C

All intensity data were log transformed before further analysis. The proteomics intensity data were tested for normality (Shapiro-Wilk) and equal variance (t-test for homogeneity of variance for two dependent samples), and if data were normally distributed, the treatment effect was analyzed by a paired Student's t-test. Non-normally distributed data were analyzed by Wilcoxon signed rank test.

SigmaPlot 11.0 was used for both statistical analysis and graphical presentation in all studies.

## Results

Skeletal muscle biopsies from 3 protocols were included: two “acute” studies (A and B), and one “prolonged study (C). Study A included serial muscle biopsies 2–10 hours following a single i.v. injection of rHuEpo (15.000 IU) or placebo in a non-fasting state. Study B was included to provide a muscle biopsy in the fasting state already 60 min after a single i.v. injection of rHuEpo using a higher dose (∼30.000 IU) or placebo. In study C the subjects were treated with rHuEpo (5000 IU) s.c. every second day for 16 days, and biopsies were collected at baseline and after the last injection. Thus, study A and B were performed in order to elucidate the acute and direct effects of rHuEpo treatment on Epo-R signalling, while study C was used to screen for long-term effects of rHuEpo treatment in skeletal muscle.

### Plasma hormone levels

Insulin levels were significantly decreased at 4 h compared to baseline in study A in both groups and at 10 h in the rHuEpo group, reflecting a postprandial increase in insulin in response to the breakfast served at baseline. Insulin levels were also significantly increased at 10 h in the placebo compared to rHuEpo group. In study B insulin levels were similar 4 h after placebo as well as rHuEpo treatment. No significant difference in plasma levels of GH was found in either study (Table 1).

**Table 1:** Serum hormone levels.

Study A	Placebo			EPO			Interaction
	Pre	4 h post	10 h post	Pre	4 h post	10 h post	p-value
**Insulin (pmol/l)**	138.4±41.2	34.8±12.7§ *	106.5±25.5**	128.7±31.9	37.0±13.8§	76.2±20.0§	0.003
**GH (ng/ml)**	0.33±0.05	3.64±1.91	0.42±0.10	2.43±2.12	1.44±1.11	0.31±0.02	0.698

Study B	Placebo	EPO
	4 h post	4 h post
**Insulin (pmol/l)**	30.8±5.3	39.2±9.1
**GH (ng/ml)**	2.43±0.81	3.55±1.48

§ significantly different from pre (p<0.001).

*significantly different from 10 h post (p<0.001).

**significantly different from EPO 10 h post (p<0.001).

### Epo receptor

**Figure 1:**
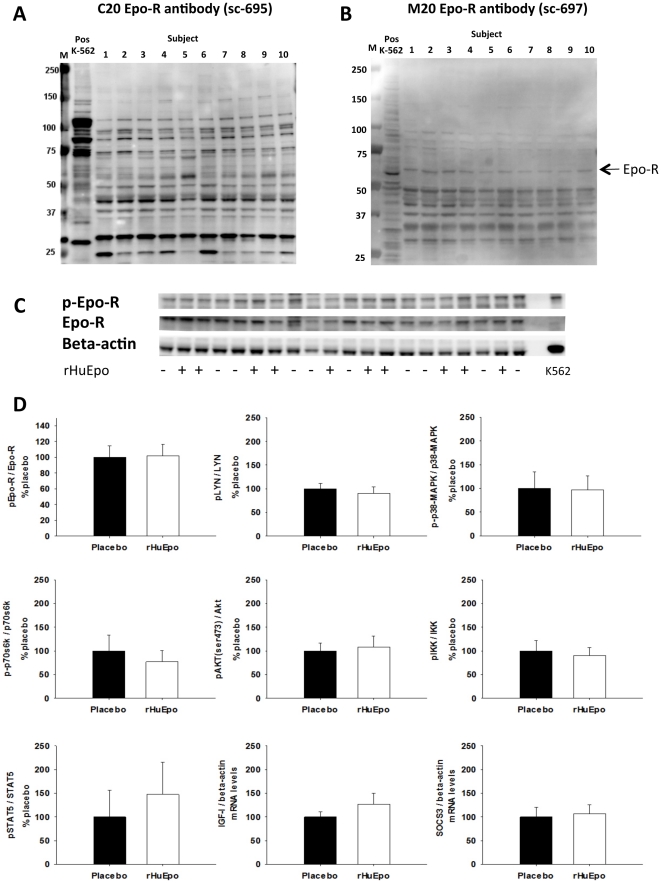
Study B: Western blot and PCR results. A+B: Identification of the Epo-R in skeletal muscle tissue in the non-stimulated state in all 10 subjects with the C20 (A) and M20 (B) antibodies. Positive control is the k-562 cells. M is the molecular marker. C: Activation of the Epo-R by phosphorylation evaluated by western blotting. The samples from the same subject in the stimulated and un-stimulated situation are loaded next to each other D: Phosphorylation of Epo-R, STAT5, p38-MAPK, Akt, p70S6 kinase, Lyn, and IKK, all normalized to the levels of the given protein. The mRNA levels of SOCS3 and IGF-I are normalized to beta-actin mRNA content. Black bars: placebo, white bars: rHuEpo. All results are from biopsies taken 1 h post treatment with either placebo or rHuEpo.

In study B, western blotting was performed with two different antibodies against the Epo-R (C20 and M20). A band, corresponding to the ∼59 kDa Epo-R, was identified in all samples and the positive control with the M20 antibody but not with the C20 antibody. Figure 1 (A+B) shows the results for the biopsy in the non-stimulated situation for all subjects compared to the positive control (k-562 cells).

Furthermore, activation of the Epo-R by phosphorylation was evaluated in both study A and B. Epo-R phosphorylation was found to be unaffected by rHuEpo administration in both studies (figure 1 C+D and figure 1).

### Signal transduction pathways

Activation of the different signalling pathways related to the Epo-R (and Epo-R phosphorylation, see above) was evaluated by western blot analysis. Beta-actin was used as loading control. The levels of β-actin protein were constant in all samples (data not shown); therefore, the level of phosphorylation was normalized only to the total level of the given protein. The membranes were stripped, hence, the same membranes were incubated with first phospho- then the total antibodies.

**Figure 2:**
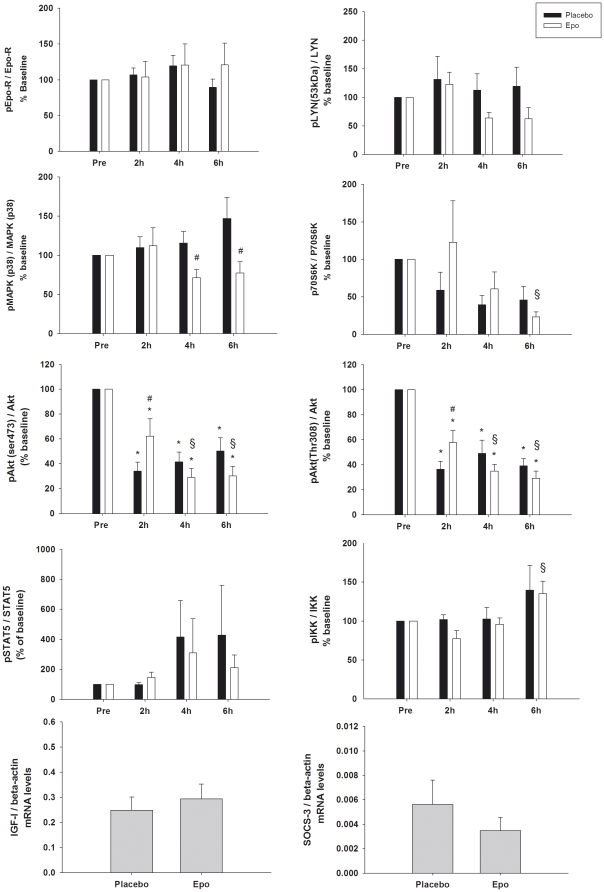
Study A: Western blot and PCR results. Phosphorylation of STAT5, p38-MAPK, Akt, p70S6 kinase, Lyn, and IKK, all normalized to the total levels of the given protein. Black bars: placebo, white bars: rHuEpo. The levels of mRNA are measured at 10 h post rHuEpo administration, and are normalized to beta-actin mRNA levels. Level of significance p<0.05, * compared to baseline, # compared to control (same timepoint), § compared to rHuEpo 2 h. The interactions were as follows; pEpo-R p = 0.318, p38-MAPK p = 0.058 (treatment effect p = 0.030), pSTAT5 p = 0.562, p-Akt(ser) p = 0.001, pAkt(thr) p = 0.007, p-IKK p = 0.742 (time effect p = 0.017), p-LYN p = 0.427, p-p70S6kinase p = 0.164 (time effect p = 0.033).

In study A, biopsies obtained before, 2 h, 4 h, and 6 h post rHuEpo administration were analysed. No significant increases in the phosphorylation of the activating sites on Lyn, STAT5, p38-MAPK, IKK, or p70S6-kinase were found after rHuEpo treatment relative to placebo (p>0.05). Akt phosphorylation was high in the first muscle biopsy in both the rHuEpo and placebo situation. This biopsy was obtained prior to rHuEpo/placebo injection but after a breakfast meal (Figure 2). Subsequent to this, the level of phosphorylation decreased after both rHuEpo and placebo injections, even though the decline after 2 h was significantly lower after rHuEpo as compared to placebo (p = 0.014 Akt(ser473) and p = 0.032 Akt(thr308)) (Figure 2). Overall, the observed pattern in Akt phosphorylation is most likely a response to elevated insulin levels (Table 1) in response to the breakfast served prior to the first biopsy, since insulin is known to be a potent activator of Akt phosphorylation. Surprisingly, MAPK phosphorylation was decreased at 4 and 6 hours post rHuEpo (4 h; p = 0.046, 6 h; p = 0.003) (Figure 2). Furthermore, sporadic increases in STAT5 phosphorylation were found. These increases may be explained by sporadic peaks in serum GH, which are known to induce activation of STAT5 in human muscle [23].

In study B, western blot analysis was performed on biopsies obtained 1 hour after rHuEpo/placebo administration in the fasting condition. Neither p-STAT5, p-MAPK, p-Akt, p-p70S6K, p-IKK or p-LYN levels were significantly different between the two treatments (p>0.05) (Figure 1).

### mRNA levels of SOCS3 and IGF-I

All mRNA results were normalised to beta-actin levels. In study B, mRNA levels were measured in the biopsies taken 1 h after treatment. Because of limited amounts of muscle tissue, mRNA levels were only measured in one biopsy 10 hours after rHuEpo administration in study A. The levels of SOCS3 mRNA did not change significantly either 1 h (study B) or 10 h (study A) post treatment (p>0.05) (Figure 1 and 2). No significant changes in IGF-I levels were found in biopsies obtained either 1 h (study B) or 10 h (study A) post treatment (p>0.05) (Figure 1 and 2).

### Muscle proteome analysis

**Figure 3:**
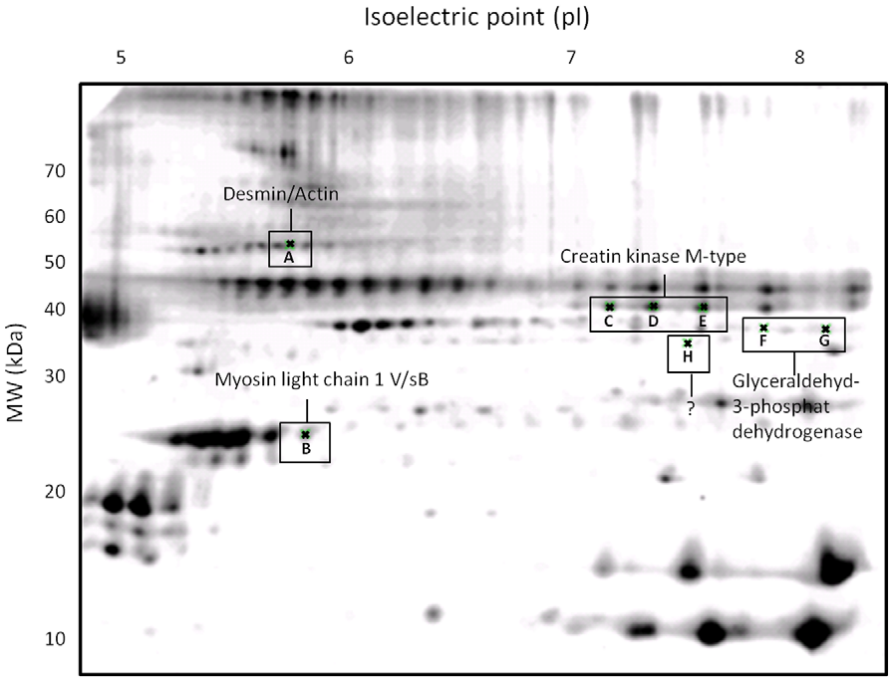
Representative 2D-gel. Representative 2D-gel of human skeletal muscle tissue. Protein spots that changed significantly (p<0.05) after 16 days of treatment with rHuEpo are shown and their identity given.

The proteome patterns for skeletal muscle tissue samples obtained in study C showed marked homogeneity when resolved by 2-DE. The pattern observed was conserved in each subject before and after treatment with rHuEpo. The high molecular weight region of the gel (>70 kDa) showed low resolution and spots in this region were therefore not analysed. A total of 201 protein spots were identified in the skeletal muscle tissue of all subjects (Figure 3).

**Figure 4:**
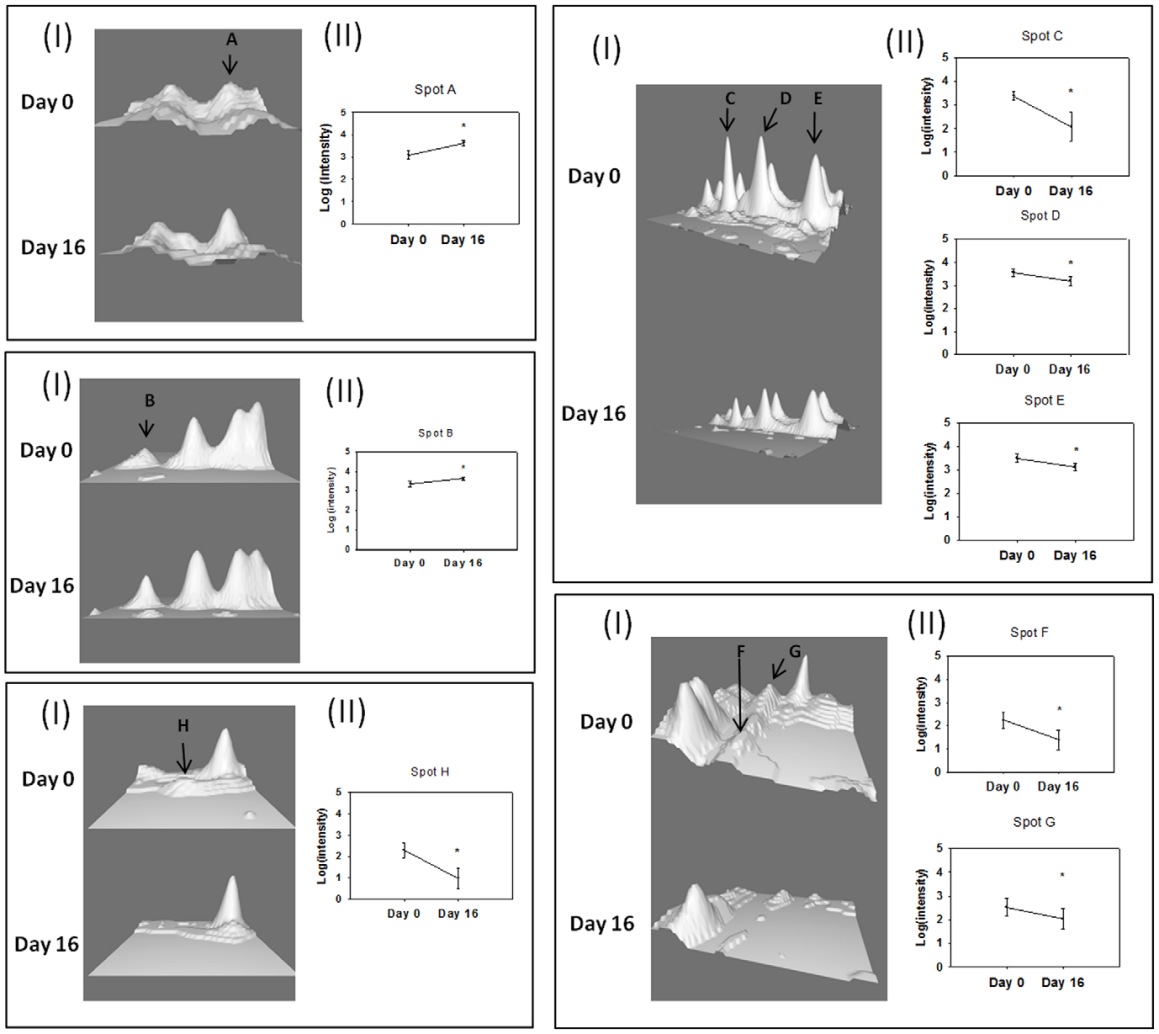
Changes in muscle proteins; Desmin/Actin (A), Myosin light chain 1 V/sB (B), Creatin kinase M-type (C, D, E), Glyceraldehyd-3-phosphat-dehydrogenase (F, G), and unidentified spot (H). I. Representative 3D image of spots showing intensity changes between baseline and day 16. Images were obtained using the 3D viewer tool from PDQuest, which converts intensity to topographical peaks. All spot images belong to the same subject. II. Mean changes in intensity for each spot. The difference between baseline and day 16 are significant (p<0.05).

Out of the 201 protein spots found in all the muscle samples, the intensity of eight spots changed significantly after 16 days of rHuEpo treatment. The intensity of two spots was increased, while the remaining six spots displayed a decrease in spot intensity. The two spots (A (p = 0.039) and B (p = 0.043)) that increased were identified by MS and tandem MS as Myosin light chain 1 V/sB (MLC1-V/sB) and a mixture of desmin and actin. Three of the spots (C (p = 0.016), D (p = 0.023), and E (p = 0.001)) that decreased were Creatine kinase M-type, and two spots (F (p = 0.031) and G (p = 0.031)) Glyceraldehyd-3-phosphate dehydrogenase (GAPDH). The intensity of the last spot (H (p = 0.047)) was very low and the identity of this spot was therefore not identified (Figure 4) (Table 2).

**Table 2:** Mass spectrometry identity matches for significant spots.

Spot #	Protein	Uniprot #		MS results			MS/MS results	
			Matched fragments	Sequence coverage (%)	Score (mascot)	Matched fragments	Sequence coverage (%)	Score (mascot)
A	Desmin	P17661	9/36	27	72	4/49	14	256
	Actin (unspecific)		5/36	17	37	2/49	7	159
B	Myosin light chain 1 V/sB	P05976	5/22	29	73	3/34	14	240
C	Creatine kinase M-type	P06732	9/18	38	121	7/29	28	338
D	Creatine kinase M-type	P06732	12/35	41	156	8/48	33	649
E	Creatine kinase M-type	P06732	15/39	49	182	7/52	33	618
F	Glyceraldehyd-3-phosphate dehydrogenase	P04406	-	-	-	1/36	4	59
G	Glyceraldehyd-3-phosphate dehydrogenase	P04406	-	-	-	2/16	8	129
H	??	-	-	-	-	-	-	-

Matched fragments; the number of peptides matched with the protein/the total amount of peptides in the sample. Sequence coverage; the percentage of the total protein that the matched peptides cover. Score; the score given by MASCOT, for MS results a score above 66 (56 for actin) was considered significant (p = 0.05), for MS/MS the significance level was a score of 34–37 (protein dependent). Mascot: http://www.matrixscience.com. Uniprot: http://www.uniprot.org.

## Discussion

This project was undertaken to study both acute and prolonged effects of systemic rHuEpo exposure in human skeletal muscle in vivo. Despite the presence of Epo-R protein by western blotting functional activity in terms of pertinent signalling proteins was absent after acute rHuEpo. By contrast, we did record unique changes in muscle protein isoform expression with prolonged treatment.

The Epo-R has previously been identified both on murine myoblasts, murine primary satellite cells [12], rat myoblasts [24], human myoblasts [24], and human skeletal muscle tissue [13], [14]. Immunohistochemical staining has localized the Epo-R to the sarcolemma [13], [14]. Epo-R mRNA and Epo-R protein have also been measured in muscle biopsies by PCR and western-blot analysis, respectively. However, the specificity of the commercially available Epo-R antibodies has been questioned [25], [26], and there is a need to develop new and more specific antibodies directed against the Epo-R. In the current study, western blotting by two different antibodies against the Epo-R was used to evaluate the presence of the Epo-R. The M20 antibody recognized a band at the level to where the Epo-R (∼59 kDa) is predicted to migrate both in the positive control and in all subjects. The band was located slightly higher than the band found in the positive control. This difference in molecular weight is most likely due to tissue specific posttranslational modifications; the positive k-562 cells are from a human leukaemia cell line in contrast to our human muscle samples. However, the C20 antibody did not detect this band. Thus, in support of the current literature [25], [26] we conclude that the C20 antibody is not able to identify the Epo-R in human skeletal muscle. Even though the M20 antibody was able to detect the ∼59 kDa band, there is literature that recommends using this antibody with caution until it has been thoroughly confirmed that this band is the Epo-R [26]. Thus, further studies are needed to confirm that this band is indeed the Epo-R.

In regard to Epo-R activation, in the current study we were not able to detect phosphorylation of the Epo-R (Tyr456). We do acknowledge that there are other phosphorylation sites on the Epo-R, which has not been evaluated here due to lack of available antibodies. In contradiction, Rundqvist et al. observed that physical activity increased phosphorylation of Epo-R associated JAK2 [14]. However, LeBaron et al. did not detect STAT5 activation after Epo stimulation to rat skeletal muscle tissue itself [27]. In support of this, Hagström et al. showed only weak amounts of Epo-R mRNA in mice skeletal muscle tissue, with no up-regulation in response to hypoxia [28]. Furthermore, Rotter et al. were not able to detect mRNA expression of Epo-R in rats under basal conditions, whereas, a transient and unspecific induction of the Epo-R gene expression was observed after traumatisation of the muscle tissue [29]. Thus, it has to be further established if the muscle tissue has to be stressed, by e.g. exercise or traumatisation, in order to express and activate the Epo-R, as shown by Rundqvist et al. [14] and Rotter et al. [29].

The presence and activation of the Epo-R in skeletal muscle tissue can be questioned, even though the presence of the Epo-R has been shown, the specificity of the antibodies used is debatable, and studies regarding Epo-R mRNA expression and activation are conflicting. It has to be established if the Epo-R is present on skeletal muscle fiber cells or only on myoblasts and satellite cells. Also, different levels of stress applied to the muscle tissue in combination with rHuEpo administration need to be evaluated in regard of Epo-R activation.

A number of different signalling molecules related to the Epo-R were analysed in the current study, including Lyn, a docking protein associated with the Epo-R in hematopoietic cells. Lyn has been shown to induce tyrosine phosphorylation of the Epo-R at levels comparable to JAK2 and to mediate activation of different signalling pathways including STAT5 [18], PI3-kinase [19], [30], IKK [20], and raf-1/MAPK [31]. However, in the present study, no change in the activation of either of the isoforms of Lyn was found upon rHuEpo administration. Likewise, no increase in the phosphorylation of STAT5, p70S6K, or MAPK was found. Akt phosphorylation in study A was strongly affected due to a postprandial increase in insulin, leading to high levels of Akt phosphorylation before the treatment with rHuEpo. Thus, the results regarding Akt in that study should be interpreted with caution. Akt phosphorylation showed a decreasing pattern over time, however this decrease was significantly attenuated 2 h after rHuEpo treatment compared to placebo in study A. Should rHuEpo stimulate Akt phosphorylation in human skeletal muscle, an increase in Akt phosphorylation after 1 h in study B would have been expected, thus, indicating that the difference seen in study A is not due to changes in the Epo levels. Surprisingly, a significant decrease in MAPK phosphorylation was observed 4 and 6 hours post rHuEpo treatment. Currently we do not have an explanation for this, but it could be indirect effects of the treatment that are induced later. Furthermore, sporadic spikes in plasma GH were observed in a few individuals, which occurred prior to transiently increased pSTAT5 levels. This emphasizes the complexity of these signalling pathways, and the importance of monitoring alternative activators of a given pathway.

One explanation for the lack of activation could be that the dose of Epo given in study A (∼15 000 IU) was too low. The dose was therefore increased to 400 IU/kg (∼32.000) in study B, which however, did not result in a detectable activation of the Epo-R. These doses are comparable to the doses used to treat patients with end-stage renal disease and stroke, respectively, and the highest dose lead to a ∼1000 fold increase in serum Epo levels. Previous studies have shown that even minimal concentrations of Epo activate STAT5, MEK, Ras, and ERKs in primary human erythroid progenitors [32], thus, we would have expected to see an activation of the signalling molecules analysed in the present studies with the relatively high doses of rHuEpo used. From the present study, however, we cannot rule out that even higher doses of rHuEpo could have activated the Epo-R.

Along the same line, it also remains to be investigated if local rather than systemic exposure to Epo would induce activation of the Epo-R in human skeletal muscle in vivo. Local exposure would also minimize the likelihood of systemic stimulation of other tissues such as the bone marrow. This could be investigated by either in situ microdialysis or local arterial perfusion. The latter approach, however, is not well suited due to the long half-life of the hormone. Microdialysis is a method that is particularly feasible for the delivery of molecules with a small molecular size. Commercially available microdialysis fibres have a cut-off value (<30 kDa) that is too low to allow diffusion of rHuEpo (30.4 kDa). It is possible to customize fibres with a higher cut-off value (3000 kDa), which would allow diffusion of rHuEpo through the membrane. From a theoretical point of view a more effective method would be electrotransfer of the Epo gene into the skeletal muscle tissue, however this method raises several ethical questions when used in humans and are therefore not applicable.

Another potential explanation for the unaltered activation of the analysed signalling pathways is that the biopsies were not taken at the appropriate time-points after rHuEpo exposure. From cell cultures it is known that Epo stimulation leads to phosphorylation of STAT5, Akt, and ERK, which peaks after 15 min and remains detectable after 60 min [33]. Probably the time for activation of these pathways in vivo will be slightly prolonged, due to transportation of the rHuEpo to the tissue of interest. It is possible that the first biopsies in study A (2 h post) were taken after maximal activation. Therefore, a biopsy was taken 1 h post rHuEpo administration in study B, which however did not reveal detectable phosphorylation of the pertinent signalling molecules. Considering that Epo has a half-life between 2–13 h [34], an up-regulation would have been expected within the time-points analysed in the present studies.

To date, no studies have been able to document robust effects of acute rHuEpo treatment in skeletal muscle. Thus, it remains a question whether the Epo-R is biologically active in skeletal muscle tissue. The results from the present study fits well with the mRNA measurements (MRF4, VEGF, HIF-1α, IGF-IEa, ferroportin, MyoD, myogen) performed by Lundby et al. on the same biopsies as analysed in study A [13]. They were not able to find a systematic regulation of a number of analysed mRNA molecules in relation to rHuEpo treatment.

Epo does however seem to affect progenitor cells of the muscle tissue (e.g. the satellite cells and myoblasts) [12], [14], [29]. Ogilvie et al. found the Epo-R to be present on these cell types and that stimulation by Epo mediates phosphorylation and thereby activation of JAK2 and to a minor extent also STAT5 [12]. Stimulation led to increased cell proliferation and decreased differentiation, which was accompanied by increased mRNA levels of MyoD and Myf5 (markers of early and mid-myogenesis) and decreased mRNA levels of myogenin (marker of irreversible commitment to terminal differentiation). Furthermore, it was shown that Epo increased the proliferation of satellite cells significantly more than placebo during the first 14 days upon muscle trauma [29]. This suggests a role for Epo in maintaining or expanding the pool of proliferating muscle progenitor cells during differentiation. This theory was partly supported by previously published results from study A, where MRF4 mRNA was transiently up-regulated 6 h after rHuEpo administration, whereas other markers of satellite cell differentiation (MyoD and myogenin) were unaltered [13]. These analyses were performed on muscle biopsies in which the abundance of satellite cells is low, which might have masked any effects of rHuEpo on these progenitor cells.

In the present study proteomic analysis was used to investigate how muscle tissue responds to prolonged treatment with rHuEpo. All the identified proteins have previously been detected in both slow and fast muscle fibres [35]. The intensity of two spots (protein isoforms) was found to be significantly up-regulated 16 days after rHuEpo administration. One spot was identified as an isoform of myosin light chain 1 V/sB (MLC1-V/sB), by MS and MS/MS analysis. The other spot corresponded to a combination of isoforms of desmin and actin. It should be emphasized that the spots, that were upregulated, belong to a train of spots (or protein isoforms), and that in the present study only one of these isoforms was significantly changed. Theoretically, these trains of spots could contain entirely different proteins with similar molecular weight and varying isoelectric points. However, each spot in a train often represents different isoforms of the same protein carrying different post-translational modifications. In the current study it was not possible by MS or MS/MS analysis to distinguish between these isoforms. The effect of Epo on myosin light chain, have been investigated by Cayla et al in rats [17]. They found that Epo treatment increases the relative amount of the slow myosin light chain (MLC) isoforms but decreases the amount of fast MLC isoforms. This change from a fast phenotype to a slower one resembles the changes seen in relation to endurance training [36]. In support of this hypothesis, proteomic analyses of slow versus fast muscle fibres showed that the slow fibres had a significant larger amount of MLC1-V/sB [35]. In the current study, MLC1-V/sB, which is the most abundant slow alkali isoform found in human muscle tissue [36], was increased which supports the findings by Cayla et al [17]. In humans, 14 weeks of rHuEpo treatment did not induce any changes in the number or size of the muscle fibres, indicating that rHuEpo does not have anabolic effects on skeletal muscle tissue [13]. Thus, further studies with longer treatment (>2 weeks) of human subjects with rHuEpo is needed to in order to clarify if rHuEpo can induce a shift from a fast glycolytic to a slow oxidative phenotype.

One spot containing desmin and actin was also significantly up regulated. Actin is a major structural protein in muscle tissue, which in combination with myosin is critical for muscle contraction. Desmin strengthens the fibrous network and connects myofibrils to each other and to the plasma membrane. The effects of Epo on these proteins in skeletal muscle are currently unknown.

The intensity of the remaining 6 spots all decreased significantly at day 16, of which the identity of one remains unidentified. Three spots were identified as creatine kinase M-type (CK) and the remaining 2 spots as isoforms of glyceraldehyd-3-phosphate dehydrogenase (GAPDH). Both of these proteins are involved in the re-synthesis of ATP. Creatine kinase reversibly catalyses the transfer of phosphate between ATP and various phosphogenes, and GAPDH is one of the enzymes involved in glycolysis and thereby in the breakdown of glucose for energy. Thus, these results could indicate that rHuEpo exerts inhibitory effects on fast generation of ATP in skeletal muscle tissue. In support of this, proteomic analyses of skeletal muscle have shown that the levels of CK and GAPDH are higher in fast muscle fibres compared to slow fibres [35]. It has previously been shown in rats, that Epo alone, without training, can induce a shift from a fast glycolytic to a slow oxidative phenotype. Epo induced a significant increase in the activity of the oxidative enzymes cytochrome c oxidase, L-3-hydroxyacyl CoA dehydrogenase and citrate synthase, however, phosphofructokinase also increased [17]. Furthermore, over-expression of Epo in mice skeletal muscle tissue lead to up-regulation of genes involved in lipid metabolism while genes involved in glucose metabolism were down-regulated [37]. In contrast, 13 weeks of treatment with rHuEpo in humans did not lead to changes in levels of either hexokinase or cytochrome c [16].

In summary, the current study supports previous findings indicating that rHuEpo treatment can lead to changes in structural proteins and various metabolic enzymes. If this ultimately leads to a shift in skeletal muscle phenotype from a fast to a slower one, still needs to be determined.

### Conclusion

The presence of the Epo-R in human skeletal muscle tissue was verified in the current study only by the M20 antibody, but we were unable to detect rHuEpo-mediated activation of the Epo-R or downstream signalling proteins in the resting condition. In contrast, by using a proteomic approach we observed changes in several isoforms of different proteins in muscle after more prolonged rHuEpo administration in a pattern compatible with increased oxidative capacity. These latter effects are likely to be indirect.
